# Experiences of work-related stress among highly stressed municipal employees in rural northern Sweden

**DOI:** 10.1080/17482631.2022.2056957

**Published:** 2022-03-29

**Authors:** Sofia Asplund, Johan Åhlin, Sture Åström, Britt-Marie Lindgren

**Affiliations:** Department of Nursing, Umeå University, Umeå, Sweden

**Keywords:** Work-related stress, occupational health, municipal employee, rural areas, suffering, qualitative content analysis

## Abstract

**Purpose:**

The aim of this qualitative study was to describe experiences of work-related stress among highly stressed municipal employees in rural northern Sweden.

**Methods:**

We interviewed 15 employees in the municipal sector in rural northern Sweden using a semi-structured guide and subjected the interviews to qualitative content analysis.

**Results:**

Under the main theme of *Suffering Through Endless Chaos*, we summarized four themes: *facing incompatible interests* and high demands due to lack of time and resources; *feeling powerless*, trapped, and ignored due to lack of control; *feeling insufficient*, insecure, and guilty due to challenging relations and high expectations; and *struggling with consequences* such as health problems, spillover effects on family life, and difficulty coping.

**Conclusion:**

Findings from this study suggest the importance of acknowledging suffering among municipal employees in a stressful work environment. An imbalance between job demands and resources is affecting both health and family lives of employees, and also their ability to work. It seems important to improve the work environment through supportive leadership, job control, and reasonable job demands to prevent stress, reduce suffering, and create a healthy organization.

## Introduction

This qualitative study focused on self-reported work-related stress among municipal employees in rural northern Sweden. Although work is generally good for employees (Waddell & Burton, 2006), and healthy workplaces provide physical, psychological, social, and organizational conditions that protect and promote health and well-being (Burton & World Health Organization, 2010), unhealthy, demanding workplaces can limit employees’ post-work recovery, impair their health, and reduce their productivity (Fernandes & Pereira, 2016). Work-related stress occurs in response to work demands that exceed employees’ ability to cope and that cannot be balanced by skills and knowledge. The World Health Organization (WHO) reports that work-related stress can be caused by poor work organization, lack of control over work, lack of support from managers and co-workers, and poor management (World Health Organization, 2020). The Job Demand-Resources (JD-R) model (Demerouti et al., 2001) is a commonly used occupational stress model, describing the impact of job demands, but also resources on occupational well-being. High job demands can exhaust employee’s mental and physical resources, and therefore lead to depletion of energy and health problems. In contrast, sufficient resources foster employee engagement and may buffer the impact of demands on stress reactions (Schaufeli & Taris, 2014). The JD-R model can be used as a framework for monitoring the workplace, aiming at increasing engagement, and preventing exhaustion (Schaufeli, 2017). Work-related stress is associated with high societal costs (Hassard et al., 2018), low work ability (Lindegård et al., 2014), various health problems (Kivimäki & Kawachi, 2015; Rose et al., 2017), and long-term sick leave (Holmgren et al., 2013). Stress that affects well-being is not always work related, but work and non-work stressors (such as private relational conflicts, caring for a family member, and financial worries (Hasselberg et al., 2014)) often combine to produce important effects. Human service occupations are at especially high risk for sick leave due to work-related stress (Aronsson et al., 2019).

Most municipal employees in Sweden work in human services such as social work, school and preschool environments, and elderly care. Most are women who often perceive their work as interesting, stimulating, and meaningful (Swedish Municipalities and Regions, 2021). However, women employed by municipalities report more health problems and are at higher risk of sick leave than men (Laaksonen et al., 2008). Such municipal employment is also associated not only with excessive workloads, psychological stress (Swedish Municipalities and Regions, 2021), but also with poorer organizational and psychosocial work environments, more health problems, higher risk of stress-related disorders, and higher sick leave rates than other occupations. The highest rates of sick leave in Sweden occur among employees in the municipal sector (Swedish Association of Local Authorities and Regions, 2019), where lack of recovery is thought to be an important link between working conditions, ill health, and sick leave (Aronsson et al., 2014). In rural areas in northern Sweden, municipal employees have among the highest rates of long-term sick leave in the country (Swedish Association of Local Authorities and Regions, 2019). Research in the northern context of Sweden has shown various health problems in the population (Eriksson et al., 2017; Klingberg et al., 2019; Lindroth et al., 2014; Norlund et al., 2010), and in the same rural context as the present study, every fifth working municipal employee reported stress-related exhaustion (Asplund et al., 2021). It seems important for employers to organize work to prevent occupational ill health among municipal employees in rural northern Sweden.

In Sweden, four out of ten occupational ill health cases reported in 2019 were caused by organizational and social factors such as high workload, limited opportunities to influence work, or problems with managers and colleagues (Swedish Work Environment Authority, 2020). According to Swedish law on work environments, employers are responsible for preventing occupational ill health and unhealthy workloads and for allocating resources so employees can meet work demands. When working conditions require, employers should also consult the Occupational Health Service (OHS) and use the support available (Swedish Work Environment Authority, 2015; Swedish Work Environment Authority, 2001). Occupational health nurses specialize in working life and therefore have an important role in assessing and managing work-related stress The Swedish municipal sector often uses the OHS for rehabilitation and individual health promotion, but only rarely for preventive and environmental workplace management (Schmidt & Sjöström, 2015).

In summary, human service occupations in the municipal sector are highly exposed to work-related stress, which is associated with a poor work environment, health problems, long-term sick leave, and high costs. Working in the municipal sector and in the rural context of northern Sweden could both contribute to more health problems, poorer organizational and psychosocial work environments, and higher sick-leave rates. Therefore, it is important to focus on understanding municipal employees’ experiences of work-related stress in order to develop measures to prevent excessive stress and keep employees healthy in their workplaces. Previous research in the area of interest is scarce, and to our knowledge, no qualitative research has focused on work-related stress among municipal employees in rural northern Sweden.

## Aim

The aim of this study was to describe experiences of work-related stress among highly stressed municipal employees in rural northern Sweden.

## Methods

To describe experiences of work-related stress among highly stressed municipal employees, we used a qualitative design to collect data through individual semi-structured interviews, which we analysed using qualitative content analysis (Graneheim et al., 2017; Graneheim & Lundman, 2004; Lindgren et al., 2020).

### Data Collection

This study was conducted in two northern rural municipalities in Sweden that were characterized by high long-term sick leave due to stress-related disorders. One municipality has about 3100 inhabitants in an area of 1600 square kilometres (618 square miles), and the other has about 12,200 inhabitants in 5500 square kilometres (2125 square miles; SCB, 2018). In the spring of 2018, municipal employees in this setting completed the Perceived Stress Scale (PSS-10; Cohen, 1983), and the Self-Rated Exhaustion (s-ED) scale (Glise et al., 2010). Those with high self-rated stress (≥20; Schwartz et al., 2016), as well as fulfilled the criteria for Self-rated Exhaustion Disorder (s-ED) were recruited to the present study. The inclusion criteria also included working in the school or nursing sector, and not being on sick leave. The 64 eligible participants were 55 women and 9 men. To ensure the greatest possible variation in the data, we used selection criteria designed to ensure final sample comprising both men and women from both municipalities and both sectors (schooling and nursing) of different ages and degrees of education. We contacted a total of 33 employees (24 women, 9 men) by sending an information letter and following up with call about a week later. Finally, we had a sample of 15 people (social workers, teachers, preschool teachers, assistant nurses, care assistants, and managers) who consented to participate in the study: 10 women and 5 men, aged 30 to 60 years (median = 42; 9 in nursing, 6 in schooling).

Individual semi-structured interviews (Brinkmann & Kvale, 2015) were carried out with 15 municipal employees at a location of their choice: their workplace, their home, or the town hall. We collected data over two months in June to August, 2018. The interviews lasted 26 to 64 (median 45) minutes. The first author performed the interviews using an interview guide with four open-ended questions: “Please describe your experiences of stress in your work life”, “Please tell me about stress in your private life”, “Please tell me of your experiences of physical and mental symptoms of stress”, and “Please tell me how you cope with stress”. Clarifying questions such as “Can you tell me more about that?” and “In what way?” allowed a richer description and deeper understanding of the interviewees’ experiences. The first author digitally recorded the interviews and a secretary, used to transcribe interviews, transcribed them verbatim.

### Data Analysis

We used inductive qualitative content analysis to interpret the textual and underlying content of the interview data (Graneheim et al., 2017; Graneheim & Lundman, 2004). We began by reading through the data several times to get an overall view of the data. We then transferred the text to the MAXQDA software program for qualitative data analysis; divided the text into meaning units (individual words, sentences, or short paragraphs relevant to the aim); condensed the meaning units to preserve their core meanings and manifest content; and labelled the meaning units with codes describing their content. Finally, before the actual analysis, we sorted the codes by their content and abstracted and interpreted them into nine subthemes, four themes, and one main theme. For example, we grouped codes such as “high blood pressure”, “sleeping problems”, “headache”, and “feeling depressed” under the subtheme *facing deteriorating health* and included it with other subthemes under the theme of *Struggling with the consequences*, which together with three other themes we interpreted under the main theme, *Suffering Endless Chaos*. The authors discussed the analysis until we reached agreement.

### Ethical Considerations

This study was approved by the Regional Ethical Review Board, Dnr 2017/495-31. Throughout the study we followed the 2013 Declaration of Helsinki recommendations to maintain human rights and adhere to scientific principles. The participants were advised, both verbally and in writing, that their participation was voluntary, their confidentiality was assured, and they could withdraw from the study at any time without giving any reason. All participants provided signed informed consent forms. Because discussing stress during the interview might evoke unpleasant feelings, participants were offered support after the interview, but none accepted the offer.

## Findings

The findings from the analysis and interpretation of the participant’s experiences of work-related stress revealed in one main theme: Suffering in the middle of an endless chaos. The findings also revealed four themes: *Facing incompatible interests, Feeling powerless, Feeling insufficient*, and *Struggling with the consequences*. Each theme consisted of two or three subthemes as presented in Table I.

**Table I.** Overview of the subthemes, themes, and main theme in the analysis

Subthemes	Themes	Main theme
Feeling overwhelmed due to lack of time	Facing incompatible interests	Suffering through endless chaos
Having limited resources		
Feeling trapped by lack of control	Feeling powerless	
Feeling ignored		
Dealing with challenging relationships	Feeling insufficient	
Feeling insecure and guilty		
Facing deteriorating health	Struggling with consequences	
Facing spillover effects on family life		
Striving to cope		

### Suffering Through Endless Chaos

The participants described suffering through endless chaos. They faced incompatible interests trying to live up to the employer’s high demands with too little time, lack of resources, and staff shortages. At the same time, the participants felt powerless in their work situation as they lacked control over both their own work and organizational changes, resulting in their feeling trapped. They also felt unsupported and ignored by management. The participants felt insufficient in challenging relations with staff and clients and insecure and guilty when they could not live up to their own high expectations. Despite, or as a result of, giving their very best in a stressful work environment, they struggled with a variety of health problems. The work-related stress spilled over into their family lives. With no energy left at the end of the workday for their family, they saw that their households suffered and they struggled to deal with stress and cope with both their work and living situations.

### Facing incompatible interests

The participants faced incompatible interests when excessive demands from their employer exceeded their ability to cope, which they described as feeling overwhelmed due to lack of time and having limited resources.

#### Feeling overwhelmed due to lack of time

The participants described feeling overwhelmed by the high demands of their employer and their lack of time to meet those demands. With too little time and an excessive workload, they had to constantly rush to complete the work tasks, which decreased the quality of their work. The stress could start at the very beginning of the work shift, when they looked over the plan and realized there was far too little time to complete the required tasks. They also lacked enough time to communicate with manager or co-workers to plan the work together. Participants often described having to skip their breaks, avoid going to the bathroom, and work overtime to be able to complete their work. The lack of time also made them prioritize certain administrative aspects of the work over the most important task, working with people. They felt unprofessional when they did not have time to help everyone because they were in a constant rushing and often running late. They avoided asking how someone was doing and made excuses to turn down offers of coffee because they had no time to sit and talk with the people who need them. No matter how much they wanted to spends even a little extra time with someone, even the shorted visit would mean even less time for the rest of the day’s work. The lack of time meant they could only do the bare minimum required. As one participant said, *“There is not time enough, with the consequence that people are suffering, and that is the most stressful thing at work.”* (IP5)

#### Having limited resources

The participants said that limited economic and human resources contributed to their inability to meet high work demands. They considered excessive workloads part of municipal employment and were stressed knowing that budget limitations prevented them from doing quality work. Participants also mentioned that working in a small municipality meant that incoming taxes, and therefore municipal budgets, were limited. Budget limitations made it difficult to hire substitutes or invest in good quality office furniture and staff uniforms. One participant said a manager had forbidden them to bring in substitutes for two years; because the sick leave rate was high, this meant that they had to work short-staffed almost every day.

*‘It is difficult for me to report sick, take time off for work, or stay home to care for my sick children.’* (IP10)

Participants also explained that the lack of substitutes willing to work in these small municipalities forced employers to hire people with no experience. Working with staff lacking the requisite knowledge, competence, or interest was reported as very stressful, especially as a manager could be responsible for over 100 workers do to the lack of qualified substitutes.

### Feeling powerless

The participants felt powerless in their work when they felt trapped by their lack of control and when they felt ignored.

#### Feeling trapped by lack of control

The participants expressed feeling trapped in their work situation because they had little freedom or control over their own work or organizational changes. Organizational changes, such as restructuring, budgetary changes, and new leadership were all reported as stressful, leading to less trust in their employer and worries about changes to or loss of their employment. They described feeling that new work assignments were out of their control. Learning about reduced expenses, creation of smaller working groups, and shifts in staffing made participants feel powerless and trapped by the employer’s decisions.

*‘It is impossible for me to influence my work situation. It is the manager who leads and distributes the work. I get angry, but it passes after a while.’* (IP13)

Participants said they lacked freedom at work, even for such simple things as planning lunch, because their workload could increase without notice. They also reported feeling helpless as they had no control over work decisions including salary, especially as there were few opportunities find a better job in a small municipality. This contributed to their feeling of being trapped in their employment. One participant said, *“It is not easy finding a new job, and at my age, it is even more challenging”* (IP8).

#### Feeling ignored

Participants described feeling ignored and abandoned by immediate and higher management. They described their immediate manager as absent, difficult to get hold of, inattentive, uncaring, or unsupportive. They also expressed feelings of loneliness, frustration, and powerlessness. The participants said that their immediate managers knew that employees often suffered stress-related ill health, but ignored the situation even when the participants cried and explained how stressed they were. Many manager blamed employees’ ill health on their private lives and offered them no support; other promised support when employees asked for their workload to be lessened, but a year later had still made no effort to offer relief. One participant said, *“I have the right to be seen and noticed, but my immediate manager chooses to close their eyes and ignore us”* (IP6).

Participants also felt the high rates of employee ill health and sick leave made the imbalance in their work organization obvious, but they said management chose to remain silent about these problems. They suggested that many employees left because they felt insignificant and ignored by management. No one in authority seemed to care about the work environment, and management seemed to think that workers were all the same, not worth valuing either as a worker or a person. One participant said, *“The management’s attitude is that we employees are interchangeable”*. (IP4)

### Feeling insufficient

Participants described feeling unable to meet both their own and their employers’ high emotional demands, dealing with challenging relationships, and feeling insecure and guilty.

#### Dealing with challenging relationships

The participants described feeling uncertain and challenged in their dealings with managers and co-workers while already managing challenging work in the human services sector. They reported feeling mentally and emotionally stressed not only by the work itself, but also by conflicts arising from different opinions about the work that could culminate in bullying and gossip from management and staff. One participant said,

*‘Just trying to get along with my co-workers can be stressful, because I’m a strong person, but I don’t want to become unfriendly with anyone*.’ (IP9)

Several participants described working in an atmosphere characterized by arguments between colleagues or with the manager. This atmosphere of intrigue and rumour resulted in some employees being relocated to other municipal workplaces and some managers quitting their jobs, which some blamed on the preponderance of women in these workplaces. The intrigues took a lot of energy, deteriorated their team work, and made it difficult for people to complete their work effectively. The small size of the municipalities also seemed to encourage this interpersonal friction. One participant said,

*‘There is too little space … everyone keeps an eye on each other. There is a culture, and it seems to be part of belonging to talk badly about each other.’* (IP3).

Participants also felt emotionally stressed when they were uncertain how best to manage and help people with demanding needs and problems such as poor mental health, language difficulties, lack of motivation, or challenging behaviours. In addition, they had to deal with parents and relatives who could often be unhappy with their work and demand they work beyond their normal tasks. They rarely got positive feedback, but were commonly scolded, which was upsetting and stressful. One participant worried, *“Will the relatives be angry with me since the communication does not work? Will they report me?” and said, “It is rare to get positive feedback from relatives”*. (IP7)

#### Feeling insecure and guilty

The participants described having a troubled conscience and feeling they were not good enough at their work. They felt guilty about not reaching high standard or when negative things happened at work, even when those things were beyond their control. Even when their managers told them they were doing a good job, they felt insufficient. They felt they were just surviving each day and not performing well enough. One participant said,

*‘I accuse myself. “What did I do wrong? What could I have done? Could I have given more support?” It is difficult letting go of these thoughts and I feel burdened.’* (IP7).

Some continued to think about what they may have left undone, blaming themselves when they did not achieve the results they expected. They felt they were not doing their very best. They also had difficulty lowering their expectations when working with people, as they experienced this as lowering the quality of their work. They wanted to do more and help more all the time, considering their work as more than *“just a job”*. Participants often felt guilty when they refused work tasks in an attempt to reduce their workload and stress. They knew that their refusal meant that someone else’s workload would be higher. Their troubled conscience often followed them home from work, which was stressful and made it difficult for them to relax in their free time. They were also stressed by uncertainty about whether their knowledge and skills were sufficient for their work. One participant said,

*‘It is stressful not knowing if I have enough knowledge to perform well at work. What do my colleagues think? I probably have to learn more’* (IP4).

### Struggling with the consequences

Participants described having to deal with the consequences of stress, including *facing deteriorating health, facing spillover effects on family life*, and *struggling to cope*.

#### Facing deteriorating health

The participants described negative effects on their physical and mental health as a consequence of their exposure to stress. Some reported feeling depressed, losing their joy in life, and not wanting to do anything. Some found themselves overly emotional and prone to crying easily: just being asked how they were feeling could induce tears. One participant said,

*‘It is awful. several staff have been hugging each other and crying because of the stress at work. This is not a good workplace.’* (IP15)

They also described having cognitive problems, such as lapses of memory. They could forget work tasks when they felt stressed or have difficulty finding words, which hindered their ability to work with people. Their sleep was also negatively affected. They could have difficulty falling asleep, wake up in the middle of the night, and/or rise too early in the morning. One participants said, *“The first thing that happens when I get stressed is that I stop sleeping”*. (IP13).

These difficulties resulted in their getting few hours’ sleep and being tired at work. Other stress-related health problems included upset stomach, neck and back pain, headache, and hypertension. Some also experienced panic attacks at work, feeling their heart beating to quickly or finding it difficult to breathe and being afraid that their heart would just stop.

#### Facing spillover effects on family life

Participants reported that giving their all in working life had negative spillover effects on their family life. By prioritizing their work, they had no energy left when they came home. One participant said, *“I just lie on the couch all evening when I get home from work, unable to do anything”* (IP12).

They also felt burdened by having to maintain their partner relationships and care for their children and animals. Some described their life as mostly survival, with only enough energy left to spend about one day a week with their children and none left for maintaining their homes, being with friends, exercising, or any of the activities they had enjoyed for years. They had no room for any stress from their private life, but they could not enjoy time at home or on holidays as they continued to spend their energy worrying about what was waiting for them when they returned to work. Stress from work was often expressed at home as impatience, frustration, and irritability. As one participant described,

*‘I take my bad mood home with me and my boys get affected and also get in a bad mood. I get upset and angry over little things. I do not recognize myself anymore’* (IP7).

The employees also said work stress contributed to relationship problems with their partner and arguments over little things they would not normally care about. It was also stressful when their partner had energy and suggested different activities, but the participants only wanted to stay at home.

#### Striving to cope

These municipal employees struggled to cope with their stress, either by trying to endure the situation or trying to change it. Some focused on taking care of their own well-being, and others sought help for stress management. Some simply resigned themselves to the stressful situation and endured the work with no stress-reduction strategy, while others chose to live in the present and not think about the future and the long-term effects of their stress at work. Strategies for those who tried to reduce their stress included gardening and being out in nature, regular exercise, and maintaining supportive relationships with co-workers and managers. Having a good relationship with the immediate manager helped some participants express and handle their stress, and some were able to ask for help in reducing their workload. One participant said,

*‘The manager calls basically every day, and I get to talk about today’s work. It feels good, relieves some stress, and makes the work situation feel more manageable’* (IP2).

Several employees who sought professional psychological support for stress management said that they would never have survived their work situation without that help. Many participants coped with the stress by changing their work situation. Some chose to work fewer hours, take more breaks, and refuse overtime work. Others planned to return to studying or to retire early. Some chose to take sick leave to escape the heavy workload, and most had at least considered quitting their job, with some were already in the process and wish to leave the human services sector. One participant said: *“I’m quitting my work. I will never be a municipal employee again”* (IP15).

## Discussion

This study was aimed to describe experiences of work-related stress among municipal employees with self-reported high stress. The main theme, S*uffering Through Endless Chaos*, summarizes our overall interpretation of the participants’ experiences of working in organizations with high demands and low resources, control, and support. Participants suffered feelings of insufficiency, insecurity, and guilt, as well as negative effects on their health and family life. Similar research also found that nurse leaders felt trapped in their work situation and experienced feelings of guilt, shame, and inadequacy due to a constant lack of resources (Rudolfsson & Flensner, 2012). Participants in that study were controlled by political and economic decisions, which made them feel powerless to influence their work situation, and suffered feelings of isolation due to lack of support from their managers. In another study, women on sick leave for stress-related exhaustion described life as an endless struggle with guilt, shame, and feelings of being lost and trapped. They experienced sick leave as a challenging and life-changing experience that made it impossible to go back to their previous life (Alsén et al., 2020). The literature has described suffering in several ways. Morse’s theory of suffering (Morse, 2001) includes the components of *enduring* and *emotional sufferin*g. Enduring is presented as an emotionless state in which suppressing feelings is central to maintaining control and not breaking down. Enduring enables a person to cope with demands or emotional situations and to do what must be done. Emotional suffering, on the other hand, is a distressed state requiring energy and the release of suppressed feelings through displays of anger, crying, or other actions in which a person more or less falls apart. According to Morse, a person can move back and forth between enduring and emotional suffering depending on the context, the support available, and their energy level. Behavioural norms are also important, as people often strive to endure in public and express emotional suffering in private. Releasing and expressing emotions is considered important for people to come to accept their circumstances, move beyond suffering, and take steps towards a better future (Morse, 2001; Morse & Carter, 1996). The participants in this study demonstrated this pattern by largely enduring their demanding work situations but expressing their feelings or breaking down, usually at home, which cost them energy and negatively affected their family life. Some participants might have begun to move beyond suffering through coping strategies such as changing their work and planning a future outside municipal employment. The suffering among municipal employees and its negative consequences on health and family life need to be recognized, and its causes better understood.

This study found that the participants *faced incompatible interests* when shortages of time, economic resources, and staff made it impossible to cope with the heavy workload and demands set by the employer. This in line with previous research among employees showing that high workloads lead to stress (Arvidsson et al., 2019; Bhui et al., 2016; Sjöberg et al., 2020; Skaalvik & Skaalvik, 2015; Vinberg & Landstad, 2013), as do long working hours and understaffing (Bhui et al., 2016). High workloads have also been associated with mental health problems among municipal home care workers in northern Sweden, especially those experiencing low social support (Sjöberg et al., 2020). In Sweden, municipal managers in rural areas have described conflicting demands, high workloads, limited resources, and stress-related ill health (Vinberg & Landstad, 2013), and human service managers have described a constant demand for speed and intensity, and commonly worked overtime, skipped breaks, and lacked time for reflection. Managers also found their role expanding, leaving them less time to provide leadership as they had to spend more time on administrative tasks and documentation (Corin & Björk, 2016). Teachers have also reported lacking resources, substitutes, and opportunities to take breaks during the workday in Sweden. High job demands and low self-efficacy were the strongest explanatory variables of burnout in a longitudinal study of teachers in Sweden. Qualitative data showed that excessive workloads and a sense of inadequacy were the two major categories of demands (Arvidsson et al., 2019). Also, teachers in Norway have described feeling very stressed by time pressure (Skaalvik & Skaalvik, 2015). The results of this study seems to be in accordance with the JD-R-model (Demerouti et al., 2001), where job demands has been associated with burnout among health and social care employees in Norway (Kaiser et al., 2020) and teachers in Finland (Hakanen et al., 2006). Public Service Motivation (PSM) determines how employees in the public sector deal with their job demands and resources, a theory related to the JD-R model. Employees with high level of PSM can better handle organizational stressors, since it serves a higher goal of helping others. However, if job demands are consistently high and job resources low, highly motivated employees can lose their psychological resources, resulting in lower PSM and leading to stress and exhaustion (Bakker, 2015). To prevent the consequences of an unhealthy work environment, it is important to make sure that there is a balance between job demands and resources in the municipal sector.

The participants in this study also *felt powerless* and trapped in their work situation as they lacked control over their own work and any organizational changes. They also described a lack of support and felt ignored by the management. This is in line with previous research showing that organizational changes, low job control, and lack of support have been associated with job insecurity, work-related stress, and stress-related exhaustion (Aronsson et al., 2017; Bhui et al., 2016; Lindeberg et al., 2011; Skaalvik & Skaalvik, 2015; Smollan, 2015). In accordance with the JD-R model (Demerouti et al., 2001), job resources including autonomy and social support were the most important factors associated to engagement and negatively related to burnout among health and social care employees in Norway (Kaiser et al., 2020). Low control over decision-making and lack of support have also been reported as reasons for leaving an employment (Astvik et al., 2020). This indicates the importance of allowing municipal employees higher control and providing them more support at work to prevent work-related stress and ill health.

This study found that the participants *felt insufficient*, insecure, and guilty when dealing with challenging relationships at work and not living up to their own expectations. Similar results showed that teachers in Sweden often felt inadequate and unable to do a good job or achieve their work goals (Arvidsson et al., 2019). Teachers in Norway described having to try to adapt their teaching to students with different abilities, disabilities, and misbehaviour as challenging and stressful (Skaalvik & Skaalvik, 2015). Previous research among Finnish municipal employees showed that psychological harassment, bullying, and injustice in the workplace were associated with stress-related exhaustion (Helkavaara et al., 2011). Caregivers also described their commitment to providing the best help possible as a cause of work-related stress. However, their commitment and sense of responsibility went beyond what was expected by the management. Although they struggled to maintain boundaries, their work often negatively affected their personal life (Czuba et al., 2019). To reduce municipal employees’ feelings of inadequacy and suffering, it seems important to have a good and supportive working climate, where they can talk openly about their stress and seek solutions.

The participants in this study *struggled with the consequences* of a stressful work environment, resulting in health problems, negative spillover effects on family life, and having to cope with stress. The reported health problems included depressive symptoms, neck and back pain, and sleep problems. Previous research among Swedish municipal employees has shown associations among work-related stress, mental and physical health problems, and difficulties with sleeping (Asplund et al., 2021; Leitaru et al., 2019; Sjöberg et al., 2020). Better health has been reported from municipal employees who experience a positive atmosphere, a sense of meaning, and feedback at work (Persson et al., 2018). This study found a negative spillover effect on participants’ family life, friends, household, and physical activity due to lack of energy. This is in line with similar results in previous research in the municipal sector (Forma, 2009; Repetti & Wang, 2017; Sandmark & Renstig, 2010; Skaalvik & Skaalvik, 2015; Tsukerman et al., 2020). Work/life imbalance has been described in people who have difficulty coping both with high demands at work and with private demands such as housework and childcare (Sandmark & Renstig, 2010). Lack of energy at the end of the workday resulted in neglect of the family, a limited social life (Skaalvik & Skaalvik, 2015), and a lack of physical activity (Vinberg & Landstad, 2013). A study in Finland showed that one third of municipal employees experienced work spillover onto family life, while only one tenth experienced family spillover onto work (Forma, 2009). In the present study, the various coping strategies described included gardening, being out in nature, being physically active, getting support at work, changing the work situation, retiring early, and quitting the job. Those coping strategies and others such as having a hobby, setting boundaries for work hours, prioritizing important parts of the work, and not working during the weekends were described as important in a study among municipal school principals (Elomaa et al., 2021). A stressful work environment has been strongly associated with intentions to take a long break from work, change jobs, or retire early (Arvidsson et al., 2019; Forma, 2009; Maneschiold & Lucaci-Maneschiold, 2021). Finding a new job has been described as a way to solve the negative spillover effect from work to family life (Forma, 2009). Municipal employees in Sweden have reported even being willing to move to another municipality to get a new job (Maneschiold & Lucaci-Maneschiold, 2021). The Swedish Work Environment Act requires employers to take all steps necessary to avoid subjecting employees to ill health (Government of Sweden, 1977). It is therefore important for employers to work on both the individual and the organizational level to prevent the negative consequences of work-related stress among municipal employees. A successful collaboration with the OHS to improve organizational and social factors in the work environment could contribute to a healthy organization. Research on interventions aimed at reducing work-related stress have showed some support that individual and organizational interventions could have an impact on reducing employee stress level and increasing wellbeing. Among elderly care nursing staff, individual interventions showed short-time improvements, while organizational and a combined approach showed long-term effects on burnout (Westermann et al., 2014). A previous Cochrane review on organizational interventions among teachers showed that changing the way teachers’ work is organized may improve wellbeing (Naghieh et al., 2015). Another Cochrane on individual and organizational interventons among helthcare workers concluded that cognitive-behavioural training, mental and physical relaxation as well as changing work schedules could reduce stress, (Ruotsalainen et al., 2015). It would be valuable to test preventive interventions in these two rural municipalities, as it may contribute to more knowledge of effects on stress-related ill-health among municipal employees in rural northern Sweden.

### Methodological discussion

In qualitative research, credibility, dependability, and transferability are concepts used to achieve trustworthiness (Graneheim et al., 2017; Graneheim & Lundman, 2004). To strengthen credibility, data were collected appropriate to the aim. We recruited only participants with high self-rated stress to ensure that we found participants well suited to discussing their experiences of stress. The interviews were conducted as soon as possible after the participants rated their life as stressful. We also recruited participants from different occupations, educations, ages, and sexes to ensure a variety of experiences and rich interview data. The participants described similar experiences of work-related stress regardless of working in school or nursing sector. Both the nursing and school sector are represented in each subtheme and theme. To achieve dependability, we tried to be aware of our own pre-understandings, all authors were involved in all steps of the analysis, and we discussed the themes and subthemes until we reached consensus. In this paper we also use representative quotes in the findings and give examples of the codes included in the subthemes. We also provide a rich presentation of the participants, context, data collection, and analysis to allow readers to evaluate the transferability of the results. We think it is reasonable to believe that the findings of this study can be transferred to other municipal employees with high stress in Sweden and other Nordic countries with similar organizational structures.

### Conclusion

Findings from this study provide important insights of municipal employee’s experiences of work-related stress in rural northern Sweden, and suggest the importance of acknowledging suffering among employees in a stressful work environment. The results of this study point towards an imbalance between job demands and resources, affecting both health and family lives of employees, and also their ability to work, resulting in struggling to cope. Therefore, it seems important that the organizations in the municipal sector have a supportive and effective leadership, and to allow employees some control over their work. Furthermore, workloads and demands should be limited and supported by adequate resources in order to prevent stress at work, and reduce suffering among employees. Future studies using qualitative methods can focus on managers` experiences working in the municipal sector in rural northern Sweden. Such research can contribute to deeper understanding of different aspects influencing employee’s occupational wellbeing.

## Data Availability

The data that support the findings of this study are available on request from the corresponding author.
